# The chemopotentiation of cisplatin by the novel bioreductive drug AQ4N

**DOI:** 10.1054/bjoc.2001.1975

**Published:** 2001-08

**Authors:** R Gallagher, C M Hughes, M M Murray, O P Friery, L H Patterson, D G Hirst, S R McKeown

**Affiliations:** Radiation Science Research Group, School of Biomedical Sciences, University of Ulster at Jordanstown, Newtownabbey, Co. Antrim, BT37 0QB, Northern Ireland; School of Pharmacy, University of London, Brunswick Square, London, WC1N 1AX, UK

**Keywords:** AQ4N + cisplatin, chemopotentiation

## Abstract

AQ4N is a bioreductive drug that can significantly enhance the anti-tumour effect of radiation and cyclophosphamide. The aim of this study was to examine the ability of AQ4N to potentiate the anti-tumour effect of cisplatin and to compare it to the chemopotentiation effect of tirapazamine. In the T50/80 murine tumour model, AQ4N (50–100 mg/kg) was administered 30 min, 2.5 or 6 h prior to cisplatin (4 mg/kg or 8 mg/kg); this produced an anti-tumour effect that was approximately 1.5 to 2 times greater than that achieved by a single 4 or 8 mg/kg dose of cisplatin. Tirapazamine (25 mg/kg) administered 2.5 h prior to cisplatin (4 mg/kg) resulted in a small increase in anti-tumour efficacy. AQ4N was also successful in enhancing the anti-tumour effect of cisplatin in the SCCVII and RIF-1 murine tumour models. This resulted in an increased cell kill of greater than 3 logs in both models; this was a greater cell kill than that observed for tirapazamine with cisplatin. Combination of cisplatin with AQ4N or tirapazamine resulted in no additional bone marrow toxicity compared to cisplatin administered alone. In conclusion, AQ4N has the potential to improve the clinical efficacy of cisplatin. © 2001 Cancer Research Campaign http://www.bjcancer.com


*British Journal of Cancer* (2001) **84**(4), 625–629
© 2001 Cancer Research Campaign
doi: 10.1054/ bjoc.2001.1975, available online at http://www.idealibrary.com on IDEAL

http://www.bjcancer.com

### The chemopotentiation of cisplatin by the novel bioreductive drug AQ4N

**R Gallagher[1], CM Hughes[1], MM Murray[1], OP Friery[1], LH Patterson[2], DG Hirst[1] and SR McKeown[1]**

[1]Radiation Science Research Group, School of Biomedical Sciences, University of Ulster at Jordanstown, Newtownabbey, Co. Antrim, Northern Ireland, BT37 0QB; [2]School of Pharmacy, University of London, Brunswick Square, London, WC1N 1AX, UK

**Summary** AQ4N is a bioreductive drug that can significantly enhance the anti-tumour effect of radiation and cyclophosphamide. The aim of this study was to examine the ability of AQ4N to potentiate the anti-tumour effect of cisplatin and to compare it to the chemopotentiation effect of tirapazamine. In the T50/80 murine tumour model, AQ4N (50–100 mg/kg) was administered 30 min, 2.5 or 6 h prior to cisplatin (4 mg/kg or 8 mg/kg); this produced an anti-tumour effect that was approximately 1.5 to 2 times greater than that achieved by a single 4 or 8 mg/kg dose of cisplatin. Tirapazamine (25 mg/kg) administered 2.5 h prior to cisplatin (4 mg/kg) resulted in a small increase in anti-tumour efficacy. AQ4N was also successful in enhancing the anti-tumour effect of cisplatin in the SCCVII and RIF-1 murine tumour models. This resulted in an increased cell kill of greater than 3 logs in both models; this was a greater cell kill than that observed for tirapazamine with cisplatin. Combination of cisplatin with AQ4N or tirapazamine resulted in no additional bone marrow toxicity compared to cisplatin administered alone. In conclusion, AQ4N has the potential to improve the clinical efficacy of cisplatin. © 2001 Cancer Research Campaign http://www.bjcancer.com

**Keywords**: AQ4N + cisplatin; chemopotentiation

It is well established that regions of low oxygen tension exist in many human tumours and this can adversely affect their response to radiotherapy, resulting in failure of local control (Overgaard et al, 1991). It has also been shown that hypoxic cells are resistant to commonly used anti-cancer drugs because they tend to be furthest from blood vessels and have slower rates of proliferation than well-oxygenated cells (Kennedy, 1987).

AQ4N (1,4-bis {[dimethylamino-N-oxide) ethyl]amino} 5,8-dihydroxy-anthracene-9,10-dione) is a bioreductive drug that is selectively toxic to hypoxic cells (Patterson, 1993; Hejmadi et al, 1996). We have shown that AQ4N enhances the anti-tumour effect of both single-dose and fractionated radiation regimens, allowing a 50% reduction in radiation dose to achieve the same anti-tumour effect (McKeown et al, 1995, 1996). More recently it has been shown that AQ4N enhances the cytotoxicity of the widely used chemotherapy agent cyclophosphamide, reducing by approximately 50% the dose of cyclophosphamide needed to achieve an equivalent anti-tumour effect. In addition there was no significant enhancement of the cytotoxicity of cyclophosphamide to the bone marrow (Friery et al, 2000). AQ4N reduction involves cytochrome P450 (cyp) mediated metabolism (Raleigh et al, 1998) as does cyclophosphamide (Gervot et al, 1999). We therefore thought it important to evaluate a second routinely used cytotoxic agent that has no known enhancement linked to cyp-mediated pathways. The most successful bioreductive drug available, tirapazamine (SR4233; 3-amino-1,2,4-benzotriazine-1,4-dioxide), is currently in use in phase II/III clinical trials in combination with, among other agents, cisplatin. We therefore have chosen to test AQ4N in combination with cisplatin in 3 murine tumours and have included tirapazamine with cisplatin as a comparator.

*Received 28 September 2000*
*Revised 14 May 2001*
*Accepted 17 May 2001*

*Correspondence to:* SR McKeown

### MATERIALS AND METHODS

### Tumour models

The T50/80 tumour is a poorly differentiated mammary carcinoma that arose in a female B6D2F1 mouse (Moore, 1988). Tumours were implanted intradermally (i.d.) on the rear dorsum of male BDF mice aged 8–12 weeks using 0.05 ml of tumour brei prepared from a donor mouse. Experiments were carried out using early passages (4–12) of the tumour. The SCCVII (mouse squamous cell carcinoma) and RIF-1 (mouse fibrosarcoma) tumours were maintained by the protocol of Twentyman et al (1980). Tumour cells ($2 \times 10^5$ cells in 0.05 ml phosphate buffered saline [PBS]) were implanted i.d. into male C3H mice aged 8–12 weeks. All animal experiments were carried out in accordance with the UK Animals (Scientific Procedures) Act 1986 and met the standards required by the UKCCCR.

### Drug preparation and administration

AQ4N (15 mg/ml) was prepared in house (Patterson et al, 1993), and made up in distilled water. Tirapazamine (1.75 mg/ml) was obtained from Sanofi Research (PA, USA) as a lyophilized powder, prepared in PBS and sonicated for 20 min to aid dissolution (McAleer et al, 1992). Cisplatin was purchased pre-dissolved in water and diluted to form a 0.5 mg/ml solution. All drugs were administered as a single intraperitoneal (i.p.) injection. The maximum tolerated doses (MTDs) of individual and combination treatments were selected using preliminary experiments and published data, where available.

### Measurement of the anti-tumour effect: tumour growth delay assay

BDF mice were treated when the T50/80 tumour reached 6.5–7.5 mm geometric mean diameter (GMD). Tumours were measured 3 times weekly and the time taken to reach double its treatment volume (VDT) was used as a measure of anti-tumour efficacy. AQ4N (50–150 mg/kg) or tirapazamine (25 mg/kg, i.e. half the MTD) were administered 30 min prior to cisplatin (4 mg/kg or 8 mg/kg, i.e. the MTD of this drug when administered alone). Weight loss was routinely monitored as an indication of normal tissue toxicity and did not fall below 15% for any drug combination. Tumour growth delay (TGD) was calculated by subtracting the mean VDT for control tumours from that obtained after drug exposure. The effect on tumour growth was assessed using the analysis of variance (ANOVA) test.

### Measurement of the anti-tumour effect: in vivo/in vitro clonogenic assay

C3H mice bearing the SCCVII or RIF-1 tumours were treated upon reaching a volume of 100–200 $mm^3$. AQ4N (100 mg/kg) was administered 30 min, 2.5 h or 6 h prior to cisplatin (4 mg/kg or 8 mg/kg) and tirapazamine (50 mg/kg) was administered 2.5 h prior to cisplatin (4 mg/kg or 8 mg/kg). Anti-tumour efficacy was measured, using the in vivo/in vitro clonogenic assay of Horsman et al (1984). In outline 24 h after treatment tumours were excised, minced and digested with an enzyme cocktail (6 mg DNase, 2 mg collagenase and 2 mg pronase per 10 ml PBS) to produce a single cell suspension. Pilot experiments were carried out by plating cells at concentrations ranging from 500 to $1 \times 10^7$. This identified the cell concentration that provided sufficient, but not confluent, colonies to allow accurate counting in individual treatment groups. Using the optimum concentrations, cells were plated in triplicate and incubated in RPMI media with 10% fetal calf serum and 1% penicillin/streptomycin at 37°C in 95% air/5% $CO_2$ for 12–14 d. Colonies were fixed and stained with 0.4% crystal violet in 70% methanol and scored by eye. Surviving fraction (SF) was calculated as the number of colonies counted, divided by the number of cells plated for a given treatment, divided by the same fraction determined for untreated control tumours. All data points represent the mean of 3 independent experiments. In each experiment, 2–3 tumours were used per treatment group so that a minimum of 6 and a maximum of 9 tumours were studied per group.

### Normal tissue–spleen colony assay

The response of normal tissue to AQ4N and tirapazamine in combination with cisplatin was evaluated in the bone marrow of BDF mice by the spleen colony assay (Till and McCulloch, 1961). Donor mice were injected i.p. with various doses and combinations of the drugs (see results for details of doses and schedules). Bone marrow cells were isolated from the femurs of the donor mice 24 h later by flushing through twice with PBS (0.7 ml). Bone marrow cells from 6 donor mice were pooled, counted and injected into the tail vein of 6 recipient BDF mice, which had previously been irradiated (24 h before) at 9 Gy. Pilot experiments had previously been carried out to determine the number of cells needed to give a measurable number of colonies per spleen; this ranged from $1 \times 10^4$–$1 \times 10^7$ cells per mouse. Mice were sacrificed 8 days later and spleens were removed, placed in Bouin's stain for 1.5 h and stored in formalin (0.4%). Spleen colonies were counted by eye. Surviving fraction was calculated as before and a Bonferoni adjustment was applied to the data to show normal distribution. Statistical significance was then determined by the unpaired Student's *t*-test.

### RESULTS

### Anti-tumour effects: T50/80

AQ4N (50–150 mg/kg) alone showed very little effect on tumour growth in the T50/80 tumour model with no difference between the 3 doses administered. Combination of AQ4N (50 mg/kg) with cisplatin (4 mg/kg) resulted in a significant ($P = 0.0001$) increase in TGD of 4.5 d compared to 1.6 d obtained with cisplatin (4 mg/kg) alone (Figure 1). When the AQ4N dose was increased to 100 mg/kg the anti-tumour effect was significantly greater ($P = 0.0001$) than the effect of cisplatin at the single higher dose of 8 mg/kg. This indicates that in the presence of AQ4N, the dose of cisplatin may be reduced by half but still produces a greater effect on the growth of the tumour. No additional improvement was noted when the AQ4N dose was increased to 150 mg/kg. Increasing the cisplatin dose to 8 mg/kg allowed a 3.2-d growth delay. This was significantly increased to 7.5–9 d ($P = 0.0001$) when given in combination with AQ4N (50–150 mg/kg).

Tirapazamine alone had little effect on tumour growth. The combination of tirapazamine (25 mg/kg) with cisplatin (4 mg/kg) resulted in a small tumour growth delay of 2.9 days, which was

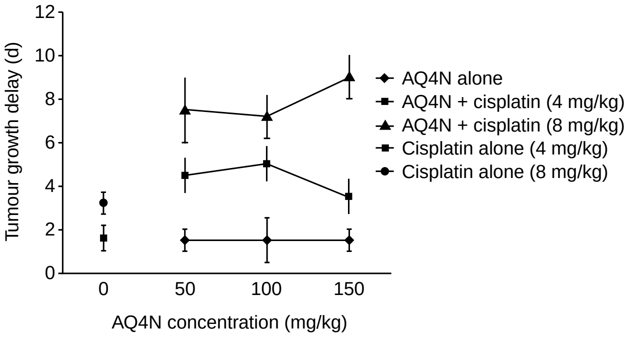

**Figure 1** Interaction of the bioreductive drugs AQ4N with cisplatin (cDDP). AQ4N (50–150 mg/kg) was administered as a single i.p. injection 30 min prior to cisplatin (4–8 mg/kg) in the T50/80 murine tumour. Results were obtained from 6–9 tumours per treatment group. Values are means ± SE

**Table 1** The anti-tumour effect of AQ4N administered at three different times prior to cisplatin in the SCCVII and RIF-1 tumour

| Treatment | Surviving Rif-1 | fraction SCCVII |
|---|---|---|
| Cisplatin (8 mg/kg) | $4.4 \times 10^{-3} \pm 0.001$ | $1.1 \times 10^{-3} \pm 0.004$ |
| AQ4N (30 min) | $< 1 \times 10^{-6}$ | $< 1 \times 10^{-6}$ |
| AQ4N (2.5 h) | $< 1 \times 10^{-6}$ | $< 1 \times 10^{-6}$ |
| AQ4N (6 h) | $< 1 \times 10^{-6}$ | $< 1 \times 10^{-6}$ |

Each drug was administered i.p. as a single dose. AQ4N (100 mg/kg) was administered 30 min, 2.5 h or 6 h prior to cisplatin (8 mg/kg). The anti-tumour effect was assessed by the clonogenic assay and the surviving fraction calculated. Results are means +/– SE obtained from three individual experiments (6–9 tumours per treatment group).

 © 2001 Cancer Research Campaign statistically greater than cisplatin administered alone (*P* = 0.038). Higher dose combinations were not possible as preliminary experiments showed unacceptable systemic toxicity beyond the doses reported here.

### Anti-tumour effects: SCCVII

Table 1 shows the pooled results (6–9 tumours) from 3 individual experiments in which AQ4N (100 mg/kg) was administered to tumour-bearing mice at various times prior to cisplatin (8 mg/kg). Figure 2 shows the results obtained when AQ4N was administered 30 min prior to cisplatin. Cisplatin at its highest dose produced over 2 logs of cell kill; this was enhanced by at least a further 4–5 logs when AQ4N was administered prior to cisplatin. The values plotted are an underestimation since this level of cell killing was at the limits of detection of the clonogenic assay. Administration of AQ4N at a range of times (30 min, 2.5 h, 6 h) prior to cisplatin had no effect on the efficacy of the drug interactions, with all 3 time schedules producing no colonies (Table 1). Tirapazamine (50 mg/-kg) administered 2.5 h prior to cisplatin (8 mg/kg) increased the anti-tumour effect by a further log in cell killing as compared to cisplatin alone.

### Anti-tumour effects: RIF-1

An 8 mg/kg dose of cisplatin administered to C3H mice bearing the RIF-1 tumour produced a cell kill of 2.5 logs (Figure 3), a slightly greater effect than observed in the SCCVII tumour. This killing was increased by at least a further 3 logs when AQ4N (100 mg/kg) was administered 30 min and 6 h prior to cisplatin (8 mg/kg) (Table 1). AQ4N (100 mg/kg) in combination with the lower dose of cisplatin (4 mg/kg) increased the cell kill by 1 log. When tirapazamine (50 mg/kg) was administered 2.5 h prior to cisplatin at doses of both 4 mg/kg and 8 mg/kg, an additional log of cell kill was obtained at the higher cisplatin dose. Both AQ4N and tirapazamine displayed very little cytotoxicity towards SCCVII and RIF-1 tumour cells when administered alone (Figure 2, Figure 3).

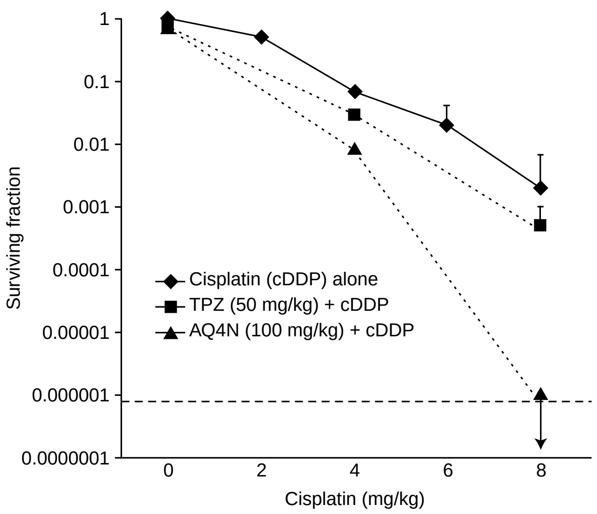

**Figure 3** Anti-tumour effect of AQ4N or tirapazamine (TPZ) in combination with cDDP in the RIF-1 model. AQ4N (100 mg/kg) was administered as a single i.p. injection 30 min prior to cDDP (4 mg/kg or 8 mg/kg). TPZ (50 mg/kg) was administered 2.5 h prior to cDDP (4 mg/kg or 8 mg/kg). Tumours were excised 24 h after treatment and clonogenic cell survival measured. Each data point represents the mean ± SE for 3 experiments (6–9 tumours per treatment group)

### Normal tissue toxicity

In order to evaluate the therapeutic potential of AQ4N, a spleen colony assay was used to determine the bone marrow toxicity of the drug when given in combination with cisplatin. From the dose response curve it can be seen that AQ4N is only slightly marrow toxic at the highest dose tested (Figure 4). Administering AQ4N

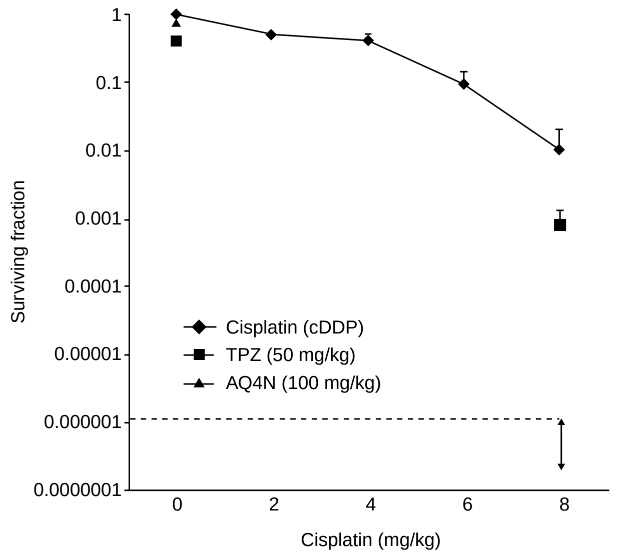

**Figure 2** Anti-tumour efficacy of the bioreductive drugs AQ4N or tirapazamine (TPZ) in combination with cDDP in the SCCVII murine tumour. AQ4N (100 mg/kg) was administered as a single i.p. injection 30 min prior to cDDP (8 mg/kg). TPZ (50 mg/kg) was administered 2.5 h prior to cDDP (8 mg/kg). Tumours were excised 24 h after treatment and clonogenic cell survival measured. Each data point represents the mean ± SE for 3 experiments (6–9 tumours per treatment group)

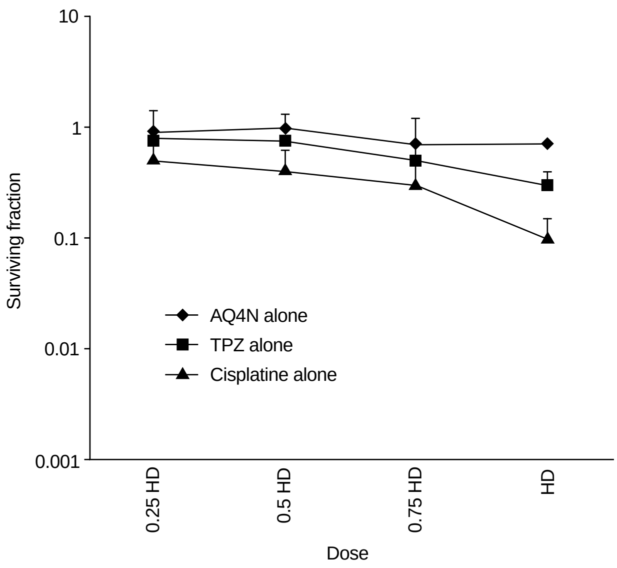

**Figure 4** The myelotoxic effect of AQ4N, TPZ and cDDP. BDF mice were dosed i.p. with AQ4N (50–100 mg/kg), TPZ (12.5–50 mg/kg) and cDDP (2–8 mg/kg). The survival of bone marrow cells was assessed by the spleen colony assay. In order to allow drug doses to be plotted on the same scale, drug doses are plotted with the highest dose used designated as HD. Results are the mean ± SE for 6 mice

*© 2001 Cancer Research Campaign*

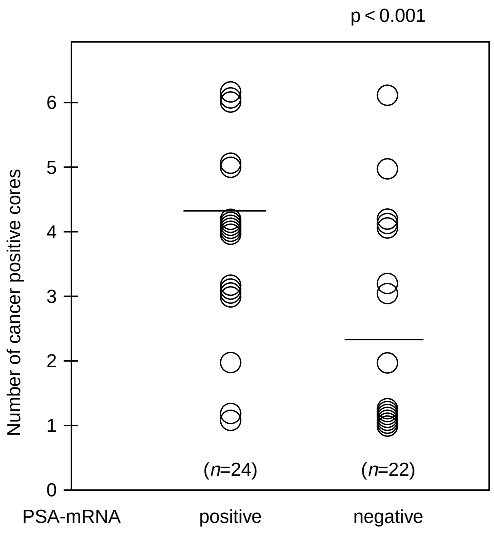

**Figure 5** The effect of AQ4N and TPZ on the bone marrow toxicity of cDDP. BDF mice were dosed i.p. with AQ4N (100 mg/kg) 30 min or 6 h prior to cDDP (4 mg/kg or 8 mg/kg). TPZ (25 mg/kg) was administered 2.5 h prior to cDDP (4 mg/kg or 8 mg/kg). The survival of bone marrow cells was assessed by the spleen colony assay. Results are mean ± SE for 6 mice

(100 mg/kg) 6 h prior to cisplatin (4 mg/kg or 8 mg/kg) did not significantly increase the toxicity caused by treatment with cisplatin alone ($P$ = 0.313 and 0.671, respectively), (Figure 4 and 5). AQ4N (100 mg/kg) given 30 min prior to cisplatin (8 mg/kg) resulted in a small increase in toxicity to bone marrow cells, although it was not statistically significant ($P$ = 0.073). Tirapazamine (25 mg/kg) alone showed a greater increase in toxicity as compared to AQ4N. However it did not potentiate cisplatin-induced bone marrow toxicity at 8 mg/kg, although a small effect was observed with 4 mg/kg ($P$ = 0.689) (Figure 5).

### DISCUSSION

Our previous studies have shown that AQ4N causes a major increase in the anti-tumour effect of cyclophosphamide in 3 tumour models, with only a limited increase in bone marrow toxicity (Friery et al, 2000). In this study the anti-tumour effect of AQ4N in combination with the chemotherapy agent cisplatin was investigated in the same 3 models, T50/80, SCCVII and RIF-1 murine tumours.

In our initial series of experiments using the T50/80 model, the combination of cisplatin (4 mg/kg or 8 mg/kg) with AQ4N resulted in an enhanced anti-tumour effect at both doses tested ($P$ = 0.0001) (Figure 1). AQ4N (50 and 100 mg/kg) with cisplatin (4 mg/kg) produced an effect ~1.5 times greater than a single 8 mg/kg dose of cisplatin alone, showing that in the presence of AQ4N, the dose of cisplatin can be reduced by 50% to give an equivalent anti-tumour effect. The combination of AQ4N with the higher cisplatin dose (8 mg/kg) produced an effect that was greater than additive with all AQ4N doses tested and was about 2 times greater than the effect achieved with cisplatin alone. Unfortunately, we could not evaluate an equivalent dose for cisplatin alone since 8 mg/kg of cisplatin is the MTD for this agent. Tirapazamine (25 mg/kg) in combination with 4 mg/kg cisplatin produced a slightly less than additive effect, although it was still equivalent to a single 8 mg/kg dose of cisplatin alone. This anti-tumour effect is probably understated since the timing schedule was not optimized for tirapazamine in this tumour model. It was also necessary to limit the tirapazamine dose in this assay to 25 mg/kg as systemic toxicity, as measured by weight loss, precluded by using higher dose combinations (data not shown).

It should be noted that cisplatin had only a small effect on T50/80 tumour growth, although this can be enhanced significantly with AQ4N. We therefore also investigated drug combinations in 2 further tumour models, including the RIF-1 tumour, which has been shown to be sensitive to combinations of tirapazamine and cisplatin (Dorie and Brown, 1993; Siemann and Hinchman, 1998). The present data demonstrates that AQ4N can significantly increase tumour cell killing observed in both the SCCVII and RIF-1 tumour models when administered prior to cisplatin (8 mg/kg). There was a surviving fraction of less than 1 in $10^6$ cells in both model systems. AQ4N alone produced few cytotoxic effects, supporting the claim that it is selectively toxic towards hypoxic cells.

Tirapazamine was administered 2.5 h prior to cisplatin since this was reported to be the optimum time schedule for this combination (Dorie and Brown, 1993). An increase of 1 log of cell kill was observed for both the SCCVII and RIF-1 model systems; this is significantly less than that reported by Dorie and Brown (1993, 1997), who showed an enhancement of 3 logs in cell kill in both models. The explanation for this discrepancy is not immediately apparent, although scheduling of tirapazamine is thought to be critical. However, our results clearly show that both bioreductive drugs can enhance the anti-tumour effect of cisplatin. Unlike tirapazamine, the enhancement of cisplatin cytotoxicity by AQ4N was not time dependent. AQ4N produced an equally impressive anti-tumour effect when given 30 min, 2.5 h, or 6 h prior to cisplatin (Table 1). This is probably due to the fact that once AQ4N undergoes enzymatic reduction to its cytotoxic metabolite, AQ4, it binds avidly to DNA (Smith et al, 1997). In contrast, Dorie and Brown have shown that the interaction between tirapazamine and cisplatin was no more than additive when the two compounds were given simultaneously and the anti-tumour effect was maximal when tirapazamine was administered 2.5 h prior to cisplatin.

The mechanism for the enhancement of the anti-tumour effect of cisplatin with either bioreductive drug can be explained in a similar way to the enhancement of radiation. Bioreductive drugs, when administered alone will kill hypoxic cells within the tumour but will have little effect on the overall tumour growth. This is only seen if a standard oxic cell cytotoxin is combined with a bioreductive agent. The advantage of killing both cell populations concurrently is clearly seen in the combination studies.

The anti-tumour effect of AQ4N in combination with cisplatin is made more impressive by the normal tissue toxicity results. The spleen colony assay was selected to test normal tissue toxicity since AQ4N and its cytotoxic metabolite, AQ4, are close analogues of the routinely used chemotherapy agent mitoxantrone (Patterson, 1993). Since one of the major sites of mitoxantrone toxicity is the bone marrow, it is not unreasonable to suggest that this might be the limiting toxicity for AQ4N. In fact, AQ4N (50–200 mg/kg) as a single agent showed very little bone marrow toxicity. In addition toxicity was less than that observed for tirapazamine, which was also modest. Cisplatin, although not considered to be a strongly myelotoxic agent, was more myelotoxic than both bioreductive drugs, with the highest dose of cisplatin resulting in a cell kill of 1 log. Siemann and Hinchman (1998), using an in vivo/in vitro bone marrow assay, reported a cell kill for cisplatin in the same range.

*© 2001 Cancer Research Campaign*

The administration of AQ4N (100 mg/kg) 6 h prior to cisplatin at both doses of 4 mg/kg and 8 mg/kg did not enhance the toxicity caused by cisplatin alone, suggesting that the 2 drugs do not have overlapping toxicities in this model. When AQ4N was administered 30 min prior to cisplatin, there was a small enhancement ($P$ = 0.073) of bone marrow toxicity at 8 mg/kg of cisplatin. This suggests that it might be advantageous to separate the administration of AQ4N and cisplatin by an interval of possibly of more than 2 h. Compared to the enhancement in the anti-tumour efficacy (> 4 logs) there was only a small enhancement of bone marrow toxicity (~0.3 logs) and this was only observed within the 30 min time schedule. AQ4N shows little schedule dependence with respect to its anti-tumour effect and thus AQ4N clearly enhances the therapeutic efficacy of cisplatin in these models. Tirapazamine (25 mg/kg) appeared to be slightly more toxic towards bone marrow cells than AQ4N. Like AQ4N, tirapazamine did not enhance the myelotoxicity of cisplatin at either dose examined, supporting the findings of Holden et al (1992) and Siemann and Hinchman (1998).

In conclusion, this study demonstrates that AQ4N can potentiate the anti-tumour effect of cisplatin. The remarkable increase in anti-tumour effect combined with the lack of bone marrow toxicity translates into a therapeutic gain. This supports the view that AQ4N is a bioreductive drug with major clinical potential, since it can enhance the anti-tumour effect of cisplatin, cyclophosphamide and radiation with little or no enhancement of bone marrow toxicity.

### ACKNOWLEDGEMENTS

This research was funded by the Ulster Cancer Foundation. The authors would like to thank Miss Wendy Allen and Miss Lynne Murphy for their technical assistance in the experimental work carried out for this paper. We would also like to thank Sanofi-Synthelabo Research, Malvern, PA, USA for the kind gift of the compound tirapazamine for use during these studies.

### REFERENCES

Dorie MJ and Brown JM (1993) Tumour-specific schedule dependent interaction between tirapazamine (SR4233) and cisplatin. *Cancer Res* **53**: 4633–4636

Dorie MJ and Brown JM (1997) Modification of the anti-tumour activity of chemotherapy drugs by the hypoxic cytotoxic agent tirapazamine. *Cancer Chemother Pharmacol* **39**: 361–366

Friery OP, Gallagher R, Murray MM, Galligan ES, Hughes CM, McIntyre IA, Patterson LH, Hirst DG and McKeown SR (2000) Enhancement of the antitumour effect of cyclophosphamide by the bioreductive drugs AQ4N and Tirapazamine. *Br J Cancer* **82**: 1469–1473

Gervot L, Rochat B, Gautier JC, Bohnenstengel F, Kroemer H, de Berardinis V, Martin H, Beaune P and de Waziers I (1999) Human CYP2B6: expression, inducibility and catalytic activities. *Pharmacogenetics* **9**: 295–306

Hejmadi MV, McKeown SR, Friery OP, McIntyre IA, Patterson LH and Hirst DG (1996) DNA damage following combination of radiation with the bioreductive drug AQ4N: possible selective toxicity to oxic and hypoxic tumour cells. *Br J Cancer* **73**: 499–505

Holden SA, Teicher BA, Ara G, Herman TS and Coleman CN (1992) Enhancement of alkylating agent activity by SR4233 in the FSaIIC murine fibrosarcoma. *J Natl Cancer Inst* **84**: 187–193

Horsman MR, Evans JW and Brown JM (1984) Enhancement of melphalan – induced tumour cell killing by misonidazole: an interaction of competing mechanisms. *Br J Cancer* **50**: 305–316

Kennedy KA (1987) Hypoxic cells as specific drug targets for chemotherapy. *Anticancer Drug Des* **2**: 181–194

McAleer JJA, McKeown SR, MacManus MP, Lappin TRJ and Bridges JM (1992) Hypobaric hypoxia: a method for testing bioreductive drugs in vivo. *Int J Radiat Oncol Biol Phys* **23**: 551–555

McKeown SR, Hejmadi MV, McIntyre IA, McAleer JJA and Patterson LH (1995) AQ4N: an alkylaminoanthraquinone N-oxide showing bioreductive potential and positive interaction with radiation in vivo. *Br J Cancer* **71**: 76–81

McKeown SR, Friery OP, McIntyre IA, Hejmadi MV, Patterson LH and Hirst DG (1996) Evidence for a therapeutic gain when AQ4N or tirapazamine is combined with radiation. *Br J Cancer* **74**: Suppl. XVII, S39–S42

Moore JV (1988) The dynamics of tumour cords in an irradiated mouse mammary carcinoma with a large hypoxic cell component. *Jpn J Cancer Res* (Gann) **79**: 236–243

Overgaard J, Grau C, Lindegaard JC and Horsman MR (1991) The potential of using hypothermia to eliminate radioresistant hypoxic cells. *Radiother Oncol* **20**: 113–116

Patterson LH (1993) Rationale for the use of aliphatic N-oxides of cytotoxic anthraquinones as prodrug DNA binding agents: a new class of bioreductive agent *Cancer Metast Rev* **12**: 119–134

Raleigh SM, Wanogho E, Burke MD, McKeown SR, Patterson LH (1998) Involvement of human cytochrome P450 (CYP) in the reductive metabolism of AQ4N, a hypoxia activated anthraquinone di-N-oxide prodrug. Xenobiotica **11**: 115–1122

Siemann DW and Hinchman CA (1998) Potentiation of cisplatin activity by the bioreductive agent tirapazamine. *Radiother Oncol* **47**: 215–220

Smith PJ, Blunt NJ, Desnoyers R, Giles Y and Patterson LH (1997) DNA topoisomerase II dependent cytotoxicity of alkylaminoanthraquinones and their N-oxides. *Cancer Chemother Pharmacol* **39**: 455–461

Till JE and McCulloch EA (1961) A direct measurement of the radiation sensitivity of normal mouse bone marrow cells. *Radiat Res* **14**: 213

Twentyman PR (1976) Comparative chemosensitivity of exponential-versus-plateau phase cells both in vitro and in vivo. *Cancer Treat Rep* **60**: 1719–1722

Twentyman PR, Brown JM, Gray JW, Franko AJ, Scoles MA and Kallman RF (1980) A new mouse tumour model system (RIF-1) for comparison of end-point studies. *J Natl Cancer Inst* **64**: 595–604

United Kingdon Co-ordinating Committee on Cancer Research (UKCCCR) (1998) Guidelines for the welfare of animals in experimental neoplasia (second edition. *Br J Cancer* **77**(1): 1–10

© 2001 Cancer Research Campaign
